# Lysosomal trafficking functions of mucolipin-1 in murine macrophages

**DOI:** 10.1186/1471-2121-8-54

**Published:** 2007-12-21

**Authors:** Eric G Thompson, Lara Schaheen, Hope Dang, Hanna Fares

**Affiliations:** 1 Department of Molecular and Cellular Biology, Life Sciences South Room 531, University of Arizona, Tucson, AZ 85721, USA

## Abstract

**Background:**

Mucolipidosis Type IV is currently characterized as a lysosomal storage disorder with defects that include corneal clouding, achlorhydria and psychomotor retardation. *MCOLN1*, the gene responsible for this disease, encodes the protein mucolipin-1 that belongs to the "Transient Receptor Potential" family of proteins and has been shown to function as a non-selective cation channel whose activity is modulated by pH. Two cell biological defects that have been described in MLIV fibroblasts are a hyperacidification of lysosomes and a delay in the exit of lipids from lysosomes.

**Results:**

We show that mucolipin-1 localizes to lysosomal compartments in RAW264.7 mouse macrophages that show subcompartmental accumulations of endocytosed molecules. Using stable RNAi clones, we show that mucolipin-1 is required for the exit of lipids from these compartments, for the transport of endocytosed molecules to terminal lysosomes, and for the transport of the Major Histocompatibility Complex II to the plasma membrane.

**Conclusion:**

Mucolipin-1 functions in the efficient exit of molecules, destined for various cellular organelles, from lysosomal compartments.

## Background

Mucolipidosis Type IV (MLIV) is a genetic neurodevelopmental and neurodegenerative disease affecting a variety of functions in patients [1-3]. A very thin corpus callosum found in MRI scans of the brain of patients indicates a deficit in embryonic brain development [1,3]. A neurodegenerative process that causes optical nerve atrophy and loss of vision occurs in all patients in childhood [4]. Patients suffer severe psychomotor retardation and most do not learn how to walk and speak. MLIV patients also have achlorhydria, or the inability to secrete gastric acid by parietal cells [5,6].

Cells in MLIV patients exhibit a number of defects. Many tissues, including the cornea, stomach parietal cells, and pancreas have large vacuoles containing fibrinogranular inclusions, multilamellar membranes and vesicles [5-10]. MLIV cells show a delay in the degradation and/or transport of endocytosed lipids that accumulate in these large vacuoles [11-15]. MLIV fibroblasts also show a defect in the fusion of lysosomes with the plasma membrane in response to treatment with the Ca^2+ ^ionophore ionomycin [16].

The gene mutated in MLIV is *MCOLN1*, which encodes mucolipin-1 (ML1) [17-19]. ML1 is predicted to have six transmembrane domains and is a group 2 Transient Receptor Potential (TRP)-related cation channel [20]. ML1 is a non-selective, pH-regulated cation channel with a preference for monovalent cations [21-24]. One possible cell biological function for ML1 in skin fibroblasts is as a proton leak channel that regulates the rate at which endosomes/lysosomes acidify [25]. ML1 localizes to late endocytic compartments and its overexpression results in abnormalities in these structures [15,22,23,26-28].

ML1 is first transported to the plasma membrane and is subsequently endocytosed and targeted to lysosomes [15,27-29]. However, the transport of ML1 is also dependent on the AP-1 adaptor complex, but not the AP-2 or the AP-3 adaptor complexes, suggesting a second direct transport route from the Trans-Golgi Network to lysosomes [30]. ML1 can be cleaved within the first intracellular loop and the two resulting portions remain associated. It is not clear whether this cleavage occurs in endosomes/lysosomes or at the Trans-Golgi Network, and whether it is required for the inactivation of the protein or is part of its normal processing [22,30].

There are two other mucolipins in mammals. Mucolipin-1, mucolipin-2, and mucolipin-3 interact to form homo- and hetero-multimers [27]. All three proteins localize to late endosomes/lysosomes, though the localization of mucolipin-3 requires an interaction with either of the other two homologues [27]. It is therefore not known whether some of the symptoms in MLIV patients are due to the mislocalization of mucolipin-3 due to the absence of ML1. While there are no known existing mutations in mucolipin-2, *varitint-waddler *(*Va*) mice have mutations in mouse mucolipin-3 resulting in deafness and pigmentation defects [31,32]. There is likely some redundancy in function between the mucolipins since DT40 B-lymphocytes lacking ML1 do not show a pronounced lysosomal defect, while in contrast, overexpression of dominant negative forms of ML1 or of mucolipin-2 results in the large vacuole defect characteristic of MLIV cells [33].

CUP-5 is the sole *Caenorhabditis elegans *mucolipin and is required for the biogenesis of lysosomes from late endosome [34,35]. Analogous to the cellular abnormalities in MLIV, mutations in *cup-5 *result in the accumulation of large vacuoles in some cells and in embryonic lethality, mostly due to developmental/tissue degeneration defects [34,36,37]. Mucolipin function is conserved since expression of human ML1 or mucolipin-3 rescues both of these defects. Mucolipin-2 has not yet been tested.

In this study, we sought to define more accurately the sites at which ML1 functions and to identify primary defects in cells with reduced ML1 levels. We used RAW264.7 cells because, like coelomocytes of *C. elegans*, macrophages have elaborate lysosomal transport pathways that allow us to visualize intermediate steps in lysosomal transport. Here, we show that ML1 is required for dynamic late endosomal/lysosomal trafficking events in macrophages.

## Results

### Co-localization of ML1 with markers for various endocytic compartments

We made a stable RAW264.7 clone in which GFP-ML1 is expressed under the control of the CMV promoter. In these stable clones, approximately 70% of the cells express GFP-ML1. A similar GFP fusion to CUP-5 is fully functional and rescues all defects of *cup-5(null) *worms, while a similar fusion to human ML1 rescues the MLIV defects in fibroblasts [16,35].

**Figure 1:**
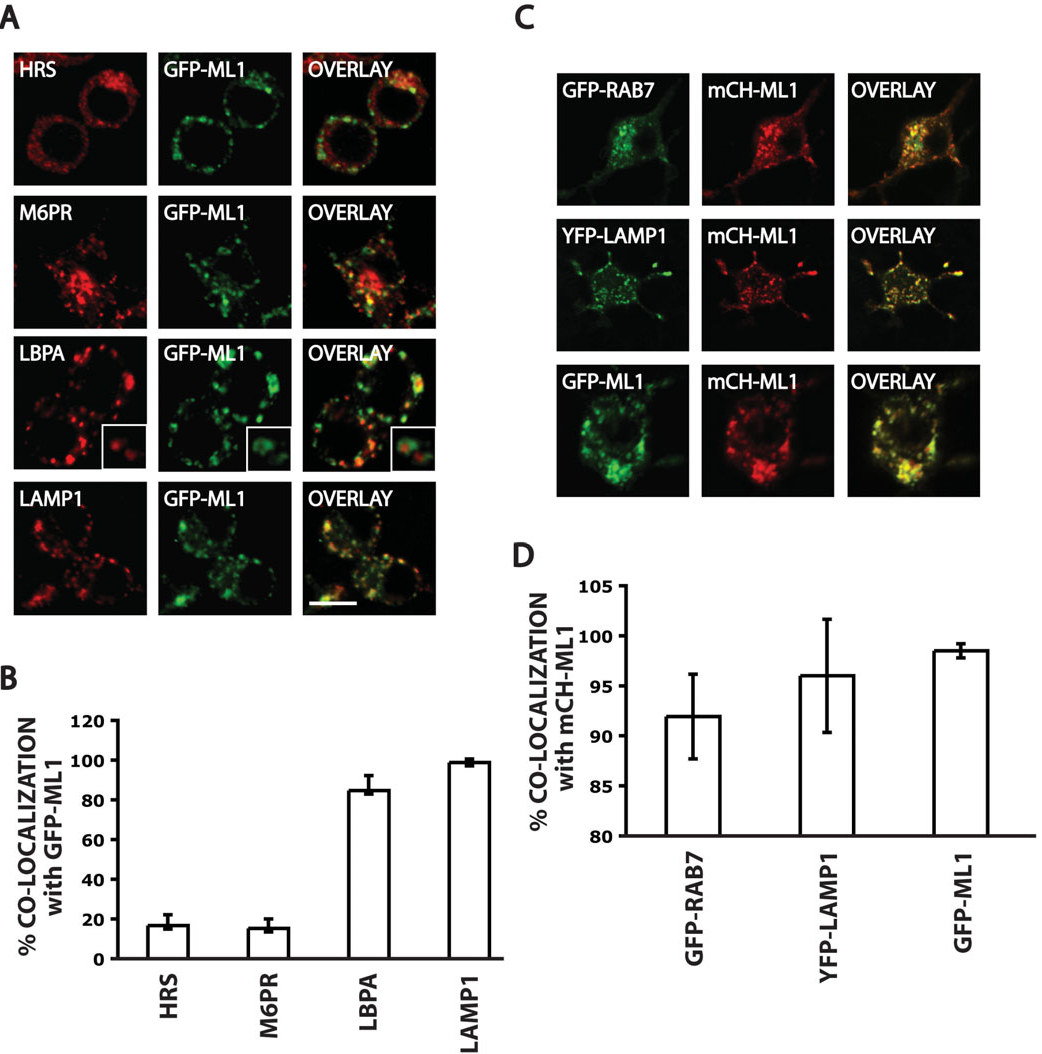
**Co-localization of ML1 with markers for various compartments**. A) Confocal images of stable GFP-ML1 (green) cells stained for the indicated markers (red). The inset in the LBPA panels is a magnification of individual compartments. Bar is 10 μm. B) Quantitation of the fraction of GFP-ML1 stainings that overlaps with each of the markers from panel A. Bars represent standard deviations. C) Confocal images of cells co-transfected with mCherry-ML1 (mCH-ML1, red) and with GFP or YFP fusions to the indicated markers (green). Bar is 10 μm. D) Quantitation of the fraction of mCherry-ML1 stainings that overlaps with each of the markers from panel C. Bars represent standard deviations.

To determine the subcellular localization of GFP-ML1, we immuno-stained these cells using antibodies against the early endosomal protein HRS, M6PR that cycles between late endosomes and the Golgi apparatus, the late endosomal/lysosomal lipid LBPA, and the lysosomal marker LAMP1 (Fig. 1A) [38-42]. Consistent with previous studies that localized ML1 to late endocytic compartments in other cell types, we saw more extensive co-localization of GFP-ML1 with late endocytic markers (Fig. 1B) [15,22,26-28]. The high incidence of co-localization of GFP-ML1 with LBPA is a novel result and indicates that ML1 localizes primarily to compartments that contain internal vesicles and lamellae [41,43].

We also made a functional fusion of the red fluorescent protein mCherry to the amino-terminus of ML1. To ascertain that this fusion protein shows the same localization pattern as GFP-ML1, we co-transfected cells with the mCherry-ML1 and the GFP-ML1 or YFP-Lamp1 expressing plasmids. Consistent with the Lamp1 immunofluorescence staining, mCherry-ML1 co-localized strongly with YFP-Lamp1 and with GFP-ML1 (Fig. 1C, D). We then co-transfected cells with mCherry-ML1 and GFP-Rab7 (late endosome/lysosome) expressing plasmids [44]. We saw significant co-localization of mCherry-ML1 with GFP-Rab7.

### Co-localization of GFP-ML1 with endocytosed substrates

**Figure 2:**
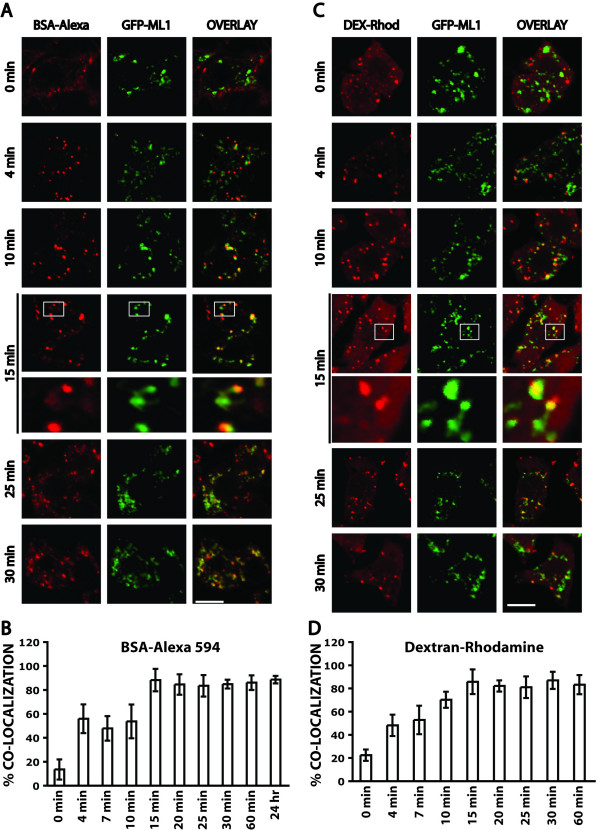
**Co-localization of GFP-ML1 with soluble endocytosed molecules**. A) Confocal images of stable GFP-ML1 (green) cells that endocytosed BSA-AlexaFluor 594 (red) for 1 minute and then chased for the indicated times before fixation. The bottom panel of the 15-minute time point represents a magnification of the area indicated in the top panel. Bar is 5 μm in unmagnified panels. B) Quantitation of the extent of co-localization of BSA-AlexaFluor 594 with GFP-ML1 at various chase times. Bars represent standard deviations. C) Confocal images of stable GFP-ML1 (green) cells that endocytosed Dextran-Rhodamine (red) for 1 minute and then chased for the indicated times before fixation. Some chase times are not shown. The bottom panel of the 15-minute time point represents an artificial magnification of the area indicated in the top panel. Bar is 5 μm in unmagnified panels. D) Quantitation of the extent of co-localization of Dextran-Rhodamine with GFP-ML1 at various chase times. Bars represent standard deviations.

To further define the localization and behavior of ML1 in lysosomal compartments, we examined the localization of GFP-ML1 relative to endocytosed soluble solutes. We pulsed stable GFP-ML1 clones with BSA-AlexaFluor 594 for 1 minute and then fixed the cells after various chase times (Fig. 2). There are four main conclusions from this analysis. First, we saw progressively increased co-localization between BSA-AlexaFluor 594 and GFP-ML1 that peaked at around 15 minutes and remained the same for 24 hours (Fig. 2B). Second, there were always some compartments that contained BSA-AlexaFluor 594 but that were not labeled with GFP-ML1, even at the 24-hour time point when all of the BSA-AlexaFluor 594 is in terminal compartments. Third, we saw many tubules emanating from GFP-ML1-labeled compartments (Fig. 2A). Fourth, we saw BSA-AlexaFluor 594 concentrations in substructures that were 300–500 nm in diameter and that were attached by tubules to endocytic compartments. GFP-ML1 localizes to these endocytic compartments, including the tubules and attached substructures (Fig. 2A, 15 min zoom). The number of these GFP-ML1-labeled compartments that showed a polarized distribution of BSA-AlexaFluor 594 increased over the time course of the experiment, peaked to about 15 +/- 2 per cell at the 15-minute time point, and remained the same at later time points. These structures were not a consequence of the overexpression of GFP-ML1 since they were apparent in untransfected RAW264.7 cells (data not shown).

We also examined the progress of the fluid-phase marker dextran-Rhodamine through GFP-ML1-labeled compartments [45]. We essentially saw the same behavior using Dextran-Rhodamine, including the extent of co-localization at various time points and the appearance of substructures with polarized distributions of Dextran-Rhodamine (Fig. 2C, D).

### Reduction of ML1 levels results in a delay in the transport of lipids to the Golgi apparatus

**Figure 3:**
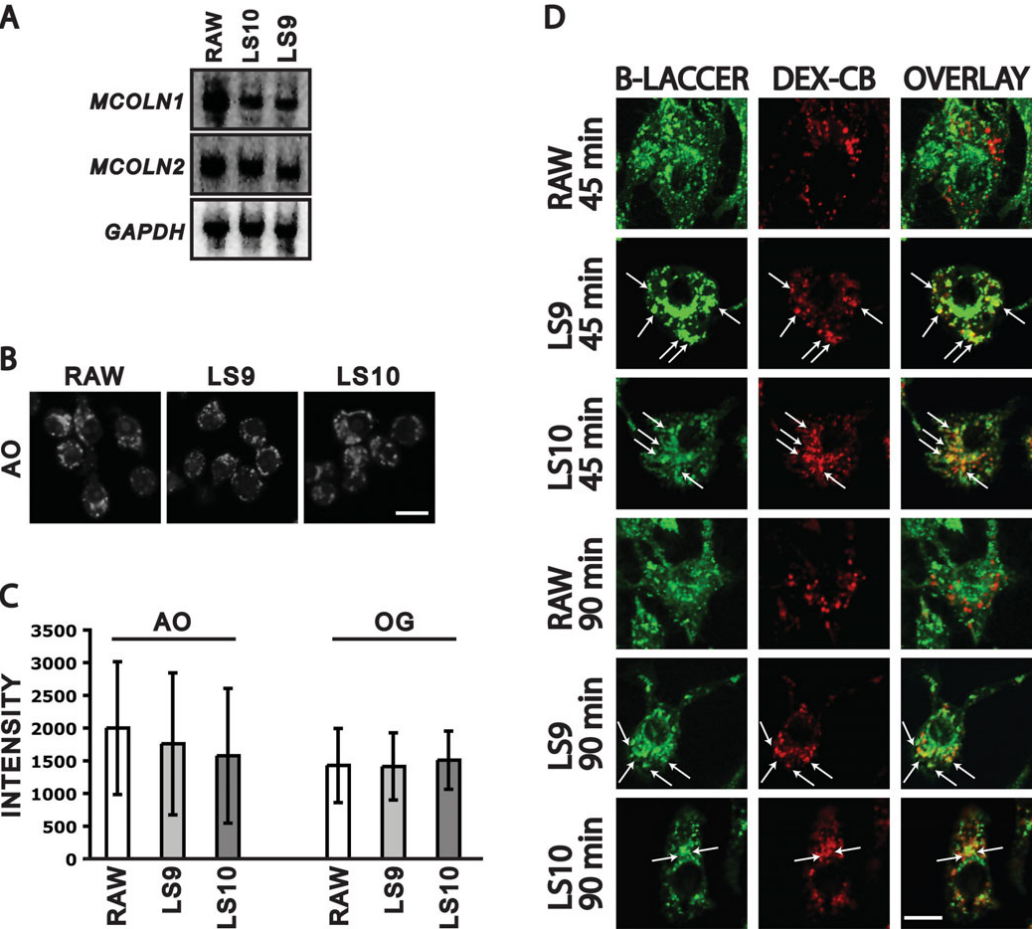
***MCOLN1 *RNAi clones**. A) Northern blot done on 15 μg of total RNA isolated from RAW264.7, LS9, or LS10 cells. The same filter was probed for *MCOLN1*, stripped, and re-probed for *GAPDH*, stripped again and re-probed for *MCOLN2*. B) Confocal images of RAW264.7, LS9, and LS10 cells stained with Acridine Orange (AO). Bar is 6 μm. C) Quantitation of the intensity of staining of AO-stained compartments and of dextran-Oregon Green (OG)-stained compartments. Bars represent standard deviations. D) Confocal images of RAW264.7, LS9, and LS10 cells whose terminal compartments were pre-loaded with Dextran-Cascade Blue (DEX-CB, red). BSA-Bodipy LacCer (B-LACCER, green) was added for 30 minutes and chased for the indicated times before fixation. Arrows indicate co-localization of the two markers. Bar is 5 μm.

We made two independently isolated RAW264.7 stable clones, called LS9 and LS10, expressing an shRNA from the constitutive histone H1 promoter and targeting *MCOLN1*. The levels of *MCOLN1 *mRNAs are significantly reduced, 19.8 +/- 3.2% and 18.7 +/- 1.7% of RAW264.7 levels in LS9 and LS10, respectively (Fig. 3A). The levels of *MCOLN2 *mRNAs are unchanged, 97.4 +/- 2.5% and 102.6 +/- 3.1% of RAW264.7 levels in LS9 and LS10, respectively (Fig. 3A). We could not detect *MCOLN3 *RNA in repeated Northern blot experiments (data not shown).

Reducing ML1 levels in RAW264.7 cells does not result in the hyperacidification of late endosomal/lysosomal compartments. This was determined either by staining of cells with the pH-sensitive dye Acridine Orange or after loading the terminal compartments with the pH-sensitive endocytic substrate dextran-Oregon Green 488 (Fig. 3B, C). Previous studies have produced conflicting data on the hyperacidification of lysosomes in MLIV fibroblasts [25,46,47]. The lack of hyperacidification of terminal compartments in our RAW264.7 RNAi lines may be due to residual ML1 activity, to redundancy with mucolipin-2, and/or the presumed pH regulatory function of ML1 may be tissue-specific.

MLIV fibroblasts also show a delay in the transport of the fluorescent lipid analogue Bodipy-LacCer from endocytic compartments to the Golgi apparatus [12,15]. To determine whether RAW264.7 cells had a similar defect, we pre-labeled the terminal compartments of RAW264.7, LS9, and LS10 cells with dextran-Cascade Blue, pulsed cells with Bodipy-LacCer for 30 minutes, and chased for 45 minutes or 90 minutes. By 45 minutes, all of the Bodipy-LacCer reached the peri-nuclear Golgi apparatus of RAW264.7 and did not co-localize with the dextran-Cascade Blue (Fig. 3D). In contrast, while some of the Bodipy-LacCer reaches the peri-nuclear Golgi apparatus in LS9 and LS10 cells, there is still significant co-localization of the Bodipy-LacCer with dextran-Cascade Blue-labeled compartments indicating a delay in the exit of Bodipy-LacCer from these compartments (Fig. 3D).

### Reduction of ML1 levels results in a delay in the transport of endocytosed proteins to lysosomes

**Figure 4:**
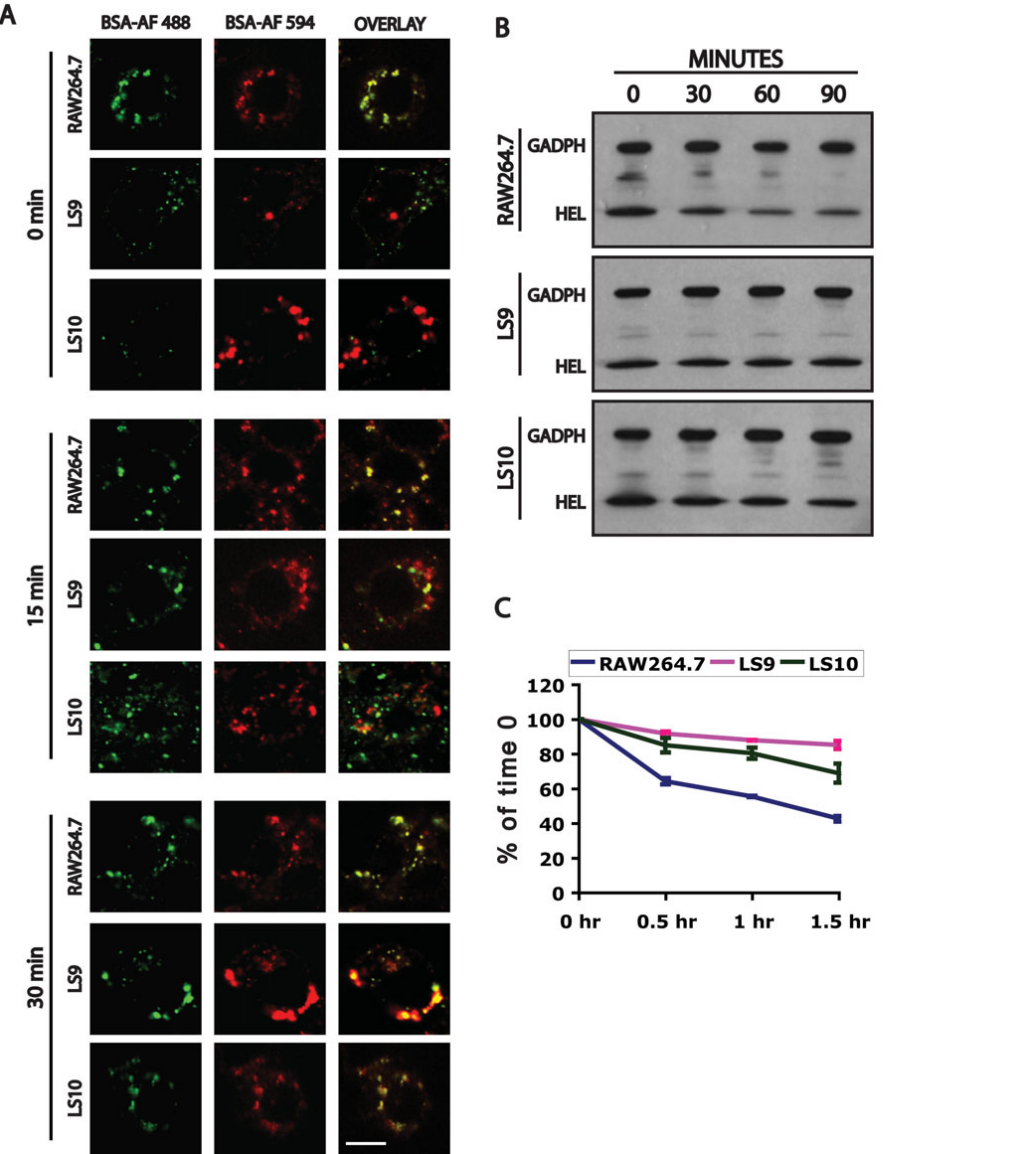
**Trafficking and endocytosis defect of proteins in *MCOLN1 *RNAi clones**. A) Confocal images of RAW264.7, LS9, and LS10 cells whose terminal compartments were pre-loaded with BSA-AlexaFluor 594 (BSA-AF 594, red). BSA-AlexaFluor 488 (BSA-AF 488, green) was added for 10 minutes to the cells and the cells were chased for the indicated times before fixation. Bar is 5 μm. B) Western blots of HEL that was endocytosed for 5 minutes and chased for the indicated times. C) Quantitation of the HEL that remains in cells relative to the 0 time point. Bars represent standard deviations from two independent assays.

Previous results had shown that CUP-5 in *C. elegans *is required for the transport of endocytosed BSA from late endosomes to lysosomes [35]. To determine whether ML1 is also required for lysosomal transport, we loaded the terminal compartments of RAW264.7, LS9 or LS10 cells with BSA-AlexaFluor 594 by incubating cells with the fluorescent marker the first day for 4 hours followed by a 24-hour incubation in the absence of the marker. Approximately 5% of LS9 and LS10 cells showed a significantly enlarged terminal compartment that contained the fluorescent endocytosed marker (Fig. 4A). In the absence of ML1-specific antibodies, we cannot determine whether these represent cells with the most reduction in ML1 levels or whether loss of ML1 in all cells would give a similarly low penetrant phenotype. The complete loss of ML1 in DT40 B lymphocytes has been reported not to affect the sizes of the terminal compartments [33].

To determine whether there is a delay in the trafficking of endocytosed proteins to terminal compartments of LS9 and LS10 cells, we pulse-chased BSA-AlexaFluor 488 into cells whose terminal compartments were pre-loaded with BSA-AlexaFluor 594 (Fig. 4A). After a 10-minute pulse, most of the BSA-AlexaFluor 488 has reached the BSA-AlexaFluor 594-stained terminal compartments in RAW264.7 cells. This co-localization in wild type cells remains the same after 15 or 30 minutes chase times (Fig. 4A). In contrast, in LS9 and LS10 cells, there is no co-localization of the BSA-AlexaFluor-488 and the BSA-AlexaFluor 594-stained compartments, either normal-sized ones or enlarged ones, after the 10-minute pulse (Fig. 4A), By 15 minutes of chase, some of the BSA-AlexaFluor 488 has reached the terminal compartments, and this co-localization is complete by 30 minutes (Fig. 4A).

Having observed a delay in lysosomal transport, we asked whether there was a delay in the degradation of endocytosed proteins. We therefore pulsed cells with Hen Egg Lysozyme (HEL) for 5 minutes and determined the remaining HEL in cells at various chase times. LS9 and LS10 cells showed increased cellular levels of endocytosed HEL at the different chase times relative to RAW264.7 cells (Fig. 4B, C). The BSA transport and HEL degradation results indicate that ML1 is required for the efficient transport of endocytosed proteins to lysosomes.

### Nature of the enlarged compartments in MCOLN1 RNAi RAW264.7 cells

**Figure 5:**
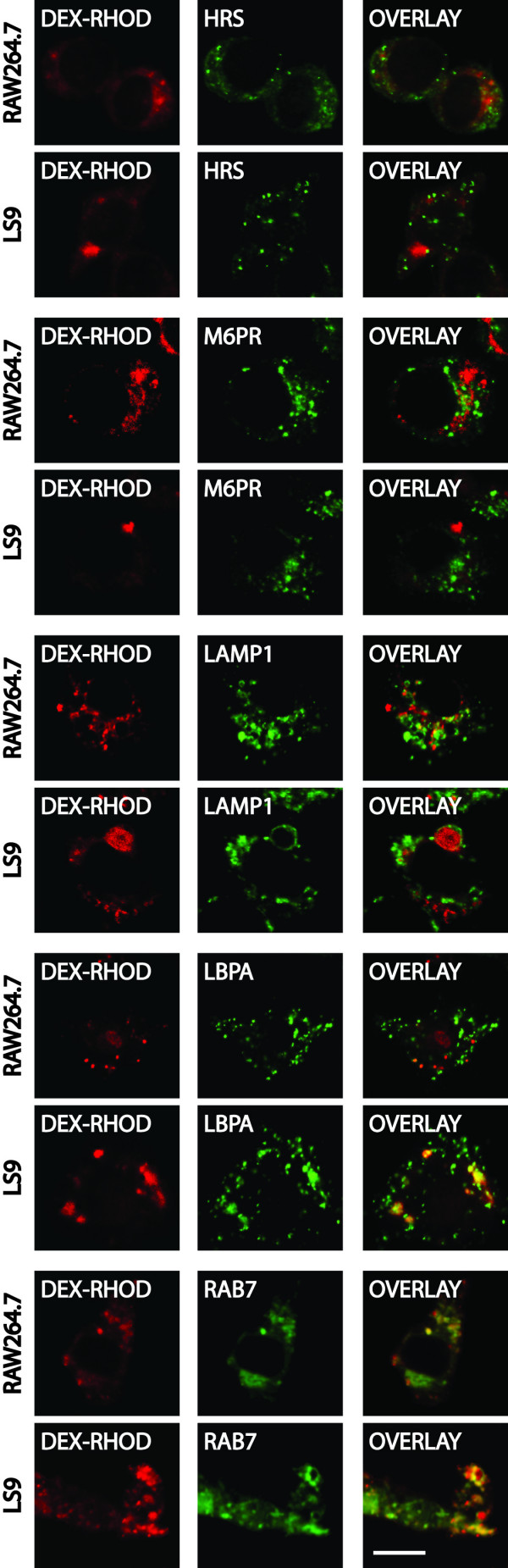
**Nature of the enlarged compartment in *MCOLN1 *RNAi clones**. Confocal images of RAW264.7 and LS9 cells whose terminal compartments were pre-loaded with Dextran-Rhodamine (red) and stained for the indicated markers (green). All of the makers were detected using immuno-specific antibodies, except for GFP-Rab7 that was transfected into wild type and *MCOLN1*- cells. Bar is 5 μm.

To determine the nature of the enlarged vacuoles in the *MCOLN1*- RNAi clones, we pre-loaded their terminal compartments with BSA-AlexaFluor 594 and stained these cells for various markers (Fig. 5). None of the enlarged vacuoles stained for M6PR or for HRS, indicating that they do not have typical early or late endosomal characteristics. These enlarged vacuoles stain for LAMP1 and for Rab7, which is consistent with their lysosomal nature. Furthermore, they strongly stain for LBPA, which is consistent with cells isolated from MLIV patients showing enlarged compartments that contain both multivesicular and multilamellar membranes [5-10].

### Reduction of ML1 levels results in a delay in the transport of MHCII to the plasma membrane

GFP-ML1 localizes to late endocytic LBPA-positive compartments and is likely required for the efficient exit of lipids and of endocytosed proteins from these compartments. The Major Histocompatibility Complex II (MHCII) localizes to LBPA-positive late endosomal/lysosomal compartments of antigen presenting cells and is transported to the plasma membrane upon stimulation of these cells [48,49]. To determine whether ML1 is required for this transport step, we first determined whether MHCII co-localizes with GFP-ML1 in normally growing cells or after addition of LPS at 100 μg/ml for one day to the cells. This LPS treatment has been previously shown to induce the differentiation of RAW264.7 macrophages into dendritic-like cells while upregulating plasma membrane levels of MHCII and of other dendritic cell surface markers [50,51].

**Figure 6:**
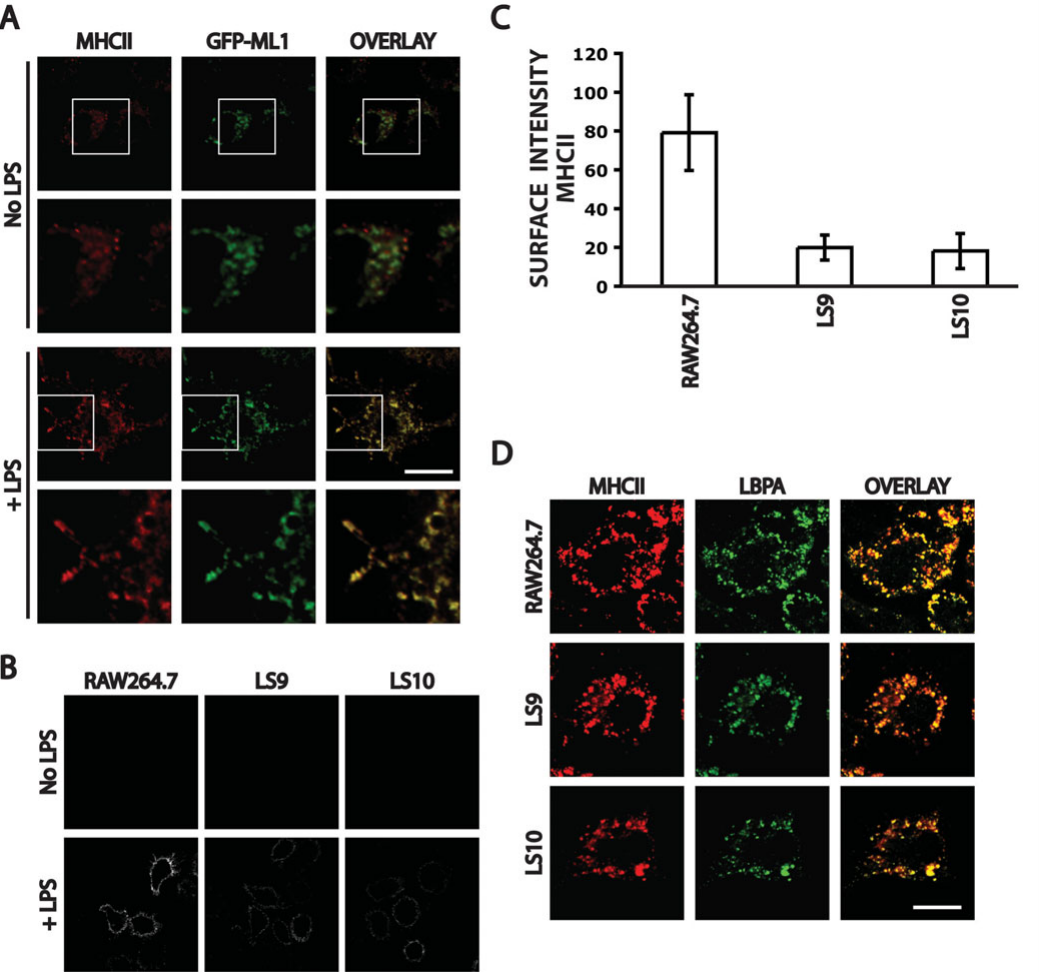
**Co-localization of GFP-ML1 with MHCII and MHCII transport to the plasma membrane**. A) Confocal images of fixed and permeabilized GFP-ML1 cells co-stained to detect MHCII (red) and GFP-ML1 (green) either in the absence of LPS or after 48 hours in 100 μg/ml LPS. Bar is 10 μm. B) Confocal images of RAW264.7, LS9, and LS10 cells stained to detect surface MHCII either in the absence of LPS or after 24 hours in 100 μg/ml LPS. All images were taken using the same exposure and magnification. C) Quantitation of the levels of MHCII at the plasma membrane in the LPS-treated cells shown in panel B. Bars represent standard deviations from at least 60 measurements for each strain. D) Confocal images of fixed and permeabilized RAW264.7, LS9, and LS10 cells co-stained to detect MHCII (red) and LBPA (green) after 24 hours in 100 μg/ml LPS. Bar is 10 μm.

In the absence of LPS, we saw some MHCII expression in GFP-ML1-positive vesicles in RAW264.7 macrophages (Fig. 6A). The addition of LPS to these cells resulted in the described increase in cell size and morphology and a dramatic enhancement of MHCII expression. In these LPS treated cells, MHCII co-localized with GFP-ML1 on tubulovesicular compartments (Fig. 6A). Because of the substantial amount of MHCII that remained in the cytoplasm of cells, we could not unambiguously visualize MHCII that had been transported to the plasma membrane.

To determine whether ML1 is required for the transport of MHCII to the plasma membrane of RAW264.7 cells, we treated RAW264.7, LS9, and LS10 cells with LPS for one days and stained cells to detect MHCII at the plasma membrane. In the absence of LPS, none of the three lines showed any surface staining. In the presence of LPS, RAW264.7, LS9, and LS10 showed plasma membrane staining, indicating that MHCII is transported to the plasma membrane in all three lines (Fig. 6B). However, the levels of MHCII at the plasma membrane of LS9 and LS10 cells were approximately four-fold lower than those of RAW264.7 cells (Fig. 6B, C). We saw the same result using two different anti-MHCII antibodies and in the presence or in the absence of IFN-γ that elevates total MHCII levels (unpublished data). RAW264.7, LS9 and LS10 cells express similar levels of intracellular MHCII as assayed by immunofluorescence staining following permeabilization (Fig. 6D). In all three lines, MHCII localizes to LBPA-positive intracellular compartments (Fig. 6D). These results indicate that in the absence of ML1, there is a reduction in the efficiency of the transport of MHCII to the plasma membrane.

## Discussion

Macrophages, like *C. elegans *coelomocytes, are highly endocytic cells. Because of the dynamic nature of lysosomal pathways in these cells, it is technically easier to characterize intermediates steps in lysosomal transport. In this study, we show that ML1 localizes primarily to LBPA-positive lysosomal compartments and is required for the efficient transport of at least two kinds of molecules from these compartments.

### ML1 localization in RAW264.7 macrophages

ML1 localizes primarily to LBPA-positive, Lamp1-positive, and Rab7-positive compartments. The limited co-localization of overexpressed GFP-ML1 with early (HRS-positive) and with late (M6PR-positive) endosomal markers is consistent with ML1 being transported to the surface and subsequently being endocytosed and transported through various endosomes before accumulating in these LBPA-positive compartments [15,27-29]. It is not known whether ML1 has separate functions in earlier steps, for example, in HRS-positive or in M6PR-positive endosomes. We think that this is unlikely because if there is a delay in trafficking from one compartment to another, then we would expect that the resulting enlarged structure would be a hybrid of these two compartments. Reducing ML1 levels results in expanded compartments that do not stain for either HRS or M6PR but that do stain for LBPA, Lamp1, and Rab7.

### Transport of endocytosed solutes in RAW264.7 macrophages

During their transport to lysosomes, endocytosed BSA and dextran are found in intermediate structures where BSA and dextran concentrate in substructures that are attached by tubules to parent compartments. GFP-ML1 localizes to all of these structures. This is strikingly similar to what has been previously observed in coelomocytes [35]. The substructures have a diameter of 300 to 500 nm, and given the time course of the experiments, likely are subcompartments that contain concentrations of lumenal proteins that are destined for lysosomes. The scission of these "buds" would segregate lysosomally-destined proteins from the rest of the LBPA-positive compartments. This is, or is analogous to, the reformation of lysosomes from hybrid organelles that has been observed both in vitro and in live cells [52,53].

While it is not yet known how BSA, dextran, and very likely other molecules are concentrated in substructures, these substructures, are topologically similar to endocytic invaginations at the plasma membrane. A possible mechanism for the concentration of endocytosed solutes in these substructures is the use of scavenger receptors that would bind lumenal molecules and cytoplasmic adaptors to concentrate these receptors in the substructures. ML1 is unlikely to have such a function since the absence of CUP-5 in worm coelomocytes does not block the concentration of BSA in substructures though it does block the scission of these from the parent compartments [35].

If GFP-ML1 is found primarily in a pre-terminal compartment, then why do we always detect a high co-incidence of localization of endocytosed molecules with GFP-ML1, even at late chase times. We think this is because terminal lysosomal compartments continuously fuse with, and deliver their content to, late endosomal compartments forming what has been termed hybrid organelles [52,53]. As mentioned above, a budding and fission reaction is used for the reformation of lysosomes [52,53].

Even at 24 hours of chase time after the uptake of fluorescent BSA, there are always some terminal compartments that contain endocytic tracers and that do not stain for GFP-ML1. We think these represent dense core lysosomes in which GFP-ML1 has been inactivated, possibly by Cathepsin B-mediated cleavage as has previously been described [22]. These dense core lysosomes continuously fuse with earlier GFP-ML1-positive compartments.

### ML1 requirement in lysosomal transport pathways

We show that reducing ML1 levels results in the delay in the transport of Bodipy-LacCer to the Golgi apparatus, of endocytosed proteins to the terminal compartments, and of MHCII to the plasma membrane. As has been previously shown, the Bodipy-LacCer co-localizes with endocytosed dextran during its transit to the Golgi apparatus, and given the high incidence of localization of dextran with GFP-ML1, very likely with GFP-ML1. Similarly, MHCII and GFP-ML1 co-localize extensively.

**Figure 7:**
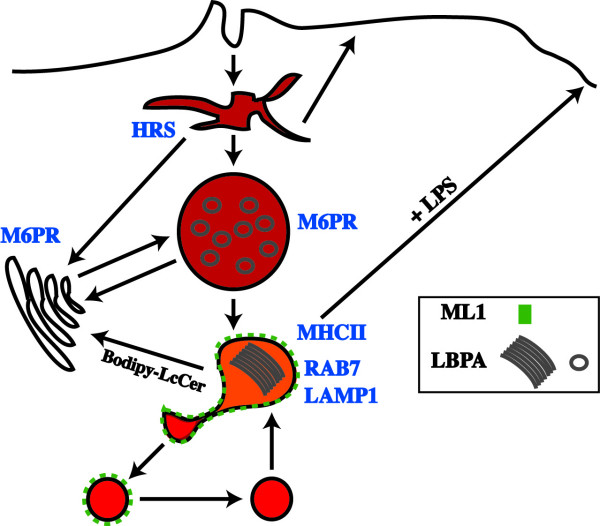
**Model of ML1 functions**. The cartoon shows the localization of ML1 (green dots) relative to soluble endocytosed molecules (shades of red). The grey circles and sheets are LBPA-stained membranes. ML1 that co-localizes with HRS and M6PR is not shown.

In our model we suggest that ML1 localizes primarily to a pre-lysosomal M6PR-negative, LBPA-positive compartment that serves as a hub for the transport of molecules to various destinations (Fig. 7). Soluble molecules like BSA and dextran are concentrated in substructures that emanate and separate from these compartments. Following the fission reaction, these substructures fuse with lysosomes, during which time ML1 is inactivated, possibly due to a reduction in pH and the activation of proteases. Previous results have implicated Cathepsin B in the proteolytic cleavage and the inhibition of ML1 channel activity in lysosomal compartments [22]. This is an iterative process in which ML1-negative lysosomes continuously fuse with GFP-ML1-labeled compartments, thus delivering partially digested peptide fragments for loading on MHCII. The transport of MHCII to the plasma membrane also involves tubulovesicular intermediates, and thus may also require ML1 for their formation or for the scission of tubules before they fuse with the plasma membrane [49].

Reducing ML1 levels results in a delay and not a block in these lysosomal transport events. In the case of BSA transport, the assays we used would only detect a delay in transport and not a complete bloc if for example ML1 is required for the formation or scission of substructures but is not required for the fusion of lysosomes with late endosomes. In addition, mucolipin-2 likely has redundant functions with ML1 since while a genomic knockout of *MCOLN1 *in DT40 B-lymphocytes does not result in expanded terminal compartments, the overexpression of dominant-negative carboxyl-terminal GFP fusions of ML1 or of mucolipin-2 both show this phenotype.

Finally, we do not know whether ML1 directly functions in Bodipy-LacCer trafficking, in BSA transport, and/or in MHCII transport. One possibility is that ML1 performs a similar function in all of these transport steps, for example, in the formation and/or scission of tubulovesicular extensions. Alternatively, ML1 may be required in one transport step such that the loss/reduction of ML1 levels retards this step leading to the accumulation of substrates in the hub compartment, and this accumulation indirectly interferes with other transport pathways. Future studies will identify specific requirements of ML1 in these transport pathways.

## Conclusion

Mucolipin-1 localizes to dynamic compartments in murine macrophages. Mucolipin-1 is required for the efficient exit of lipids destined for the Golgi apparatus, of endocytosed molecules destined for terminal lysosomes, and of MHCII destined for the plasma membrane, from these compartments.

## Methods

### Cell culture and transfection

Mouse RAW264.7 macrophages (ATCC, Manassas, VA) were grown in Dulbecco's Modified Eagle Medium (DMEM) containing 2 mM Glutamax and supplemented with 10% Fetal Bovine Serum, 100 U/ml penicillin, and 100 μg/ml streptomycin (Invitrogen, Carlsbad, CA) at 37° in 95% air at 5% carbon dioxide. LS5, the GFP-ML1 stable clone, and LS9 and LS10, the *MCOLN1 *RNAi stable clones, were grown under the same conditions and including G418 at 250 μg/ml.

Transfections of plasmids were done using Fugene 6 (Roche, Indianapolis, IN).

### Molecular methods

Standard methods were used for the manipulation of recombinant DNA [54]. Polymerase chain reaction (PCR) was done using the Expand Long Template PCR System (Roche) according to the manufacturer's instructions. All other enzymes were from New England Biolabs (Beverly, MA), unless otherwise indicated.

### Plasmids

All PCR fragments were sequenced after insertion into plasmids.

Plasmid pHD300 encoding a fusion protein of EGFP to the amino-terminus of mouse ML1 is the ~1.7 kb PCR fragment (template: mouse cDNA; primers: 5' CACACAAAGCTTATGGCCACCCCGGCGGGCCGGCGC 3' and 5' CACACAGTCGACTCAGTTCACCAGCAGCGAATGGTC 3') restriction digested with *Hind*III + *Sal*I and inserted into the same sites of pEGFP-C3 (Clontech, Mountain View, CA).

Plasmid pHD334, in which the red fluorescent protein mCherry replaces EGFP in the same frame of pEGFP-C3, was made by restriction digesting the 720 bp PCR fragment (template: pmCherry; primers: 5' CACACAACCGGTCGCCACCATGGTGAGCAAGGGCGAGGAGG 3' and 5' CACACAAGATCTGAGTACTTGTACAGCTCGTCCATGCCG 3') with *Age*I + *Bgl*II and inserting into the same sites of pEGFP-C3 [55].

Plasmid pHD339 encoding a fusion protein of mCherry to the amino-terminus of mouse ML1, was made by subcloning the ~1.7 kb *Hind*III + *Sal*I fragment from pHD300 into the same sites of pHD334.

Plasmid pHD307 expressing the mouse *MCOLN1 *shRNA was made by annealing and ligating the two complimentary oligos 84696 (5' AGCTTAAAAATCAGCCTCTTCATCTACATTCTCTTGAAATGTAGATGAAGAGGCTGAGGG 3') and 84697 (5' GATCCCCTCAGCCTCTTCATCTACATTTCAAGAGAATGTAGATGAAGAGGCTGATTTTTA 3') into *Bgl*II-*Hind*III cut pSUPER-neo (Oligoengine, Seattle, WA). This shRNA is expressed in front of the histone H1 promoter and targets the sequence 5' UCAGCCUCUUCAUCUACAU 3' in the mouse *MCOLN1 *mRNA.

### Making MCOLN1 RNAi clones

We identified two stable transfectants, LS9 and LS10, using plasmid pHD307. To determine the efficiency of the RNAi, we ran 15 μg of total RNA from RAW264.7, LS9, or LS10 cells on a gel. The Northern blot was probed with DNA fragments complimentary to *MCOLN1*, to *MCOLN2*, or to *GAPDH*. The same filter was probed for *MCOLN1*, stripped, and re-probed for *GAPDH*, stripped again and re-probed for *MCOLN2*. The intensities of the bands were quantitated using ImageJ software (N.I.H., Bethesda, MD). To determine the levels of *MCOLN1 *or *MCOLN2 *in LS9 (or LS10) relative to RAW264.7 cells, we divided the intensity of the *MCOLN1 *band by that of the *GAPDH *band in LS9 (or LS10) to get a "relative level" in each strain. The "relative level" from LS9 (or LS10) was divided by the "relative level" from RAW264.7 and multiplied by 100 to get percent change of *MCOLN1 *or *MCOLN2 *mRNA levels. The RNA isolation and Northern blots were repeated twice to calculate means and standard deviations.

### Time course of uptake: GFP-ML1

Cells that were grown on coverslips were incubated in DMEM/F-12 medium (Invitrogen) for at least 1 hour before the start of the experiment. Bovine Serum Albumin (BSA)-AlexaFluor 594 (Invitrogen) was dissolved and added to cells at 1 mg/ml in DMEM/F-12 for 1 minute at 37°. Alternatively, dextran MW 10,000-Rhodamine (Invitrogen) was added to cells at 1 mg/ml in DMEM/F-12 for 1 minute at 37°. Following the first minute of incubation, the medium was replaced with DMEM/F-12 containing 1 mg/ml BSA and the cells were fixed after various chase times at 37°. Fixation was done by adding ice-cold 2% paraformaldehyde in PBS to the cells and incubating at room temperature (RT) for 1 hr. Coverslips were washed three times with PBS before loading in Slowfade mounting medium (Invitrogen) on slides for viewing. The percent co-localization is the number of BSA-AlexaFluor 594 (or Dextran Rhodamine)-stained discrete structures that co-localized with GFP-ML1-stained structures divided by the total number of BSA-AlexaFluor 594 (or Dextran Rhodamine) stained structures in a section and multiplied by 100. The graphs show the average from sections of at least 20 different cells.

### Time course of uptake: MCOLN1-

Cells that were grown on coverslips were incubated in DMEM/F-12 medium for at least 1 hour before the start of the experiment. BSA-AlexaFluor 594 was added to cells at 1 mg/ml in DMEM/F-12 for 1 hour at 37°. The medium was replaced with regular medium and the cells were left for 24 hours to pre-label the terminal compartments. Cells were again incubated in DMEM/F-12 medium containing 2 mM glutamine for at least 1 hour before the start of the experiment. BSA-AlexaFluor 488 (Invitrogen) was added to cells at 1 mg/ml in DMEM/F-12 for 10 minutes at 37°. Cells were washed once with DMEM/F-12, incubated in DMEM/F-12 containing 1 mg/ml BSA, and the cells were fixed after various chase times at 37°. Fixation was done by adding ice-cold 2% paraformaldehyde in PBS to the cells and incubating at room temperature (RT) for 1 hour. Coverslips were washed three times with PBS before loading in Slowfade mounting medium (Invitrogen) on slides for viewing.

### Immunofluorescence

For conventional immunofluorescence, cells that were grown on coverslips were fixed for 20 minutes in 4% paraformaldehyde in PBS at RT or in 100% MetOH (kept at -20°) for 15 minutes at -20°. Cells were washed three times with PBS at RT, 5 minutes each time. Paraformaldehyde-fixed cells were incubated in 50 mM NH_4_Cl in PBS for 10 minutes at RT and washed two more times with PBS. Blocking was done for 30 min in blocking buffer (1% BSA, 0.1% Saponin, in PBS). Cells were then incubated in primary antibodies diluted in blocking buffer for two hours at RT, washed three times with PBS, incubated in Cy2 or Cy3 labeled secondary antibodies (Jackson ImmunoResearch Laboratories, West Grove, PA) diluted 1:200 in blocking buffer for one hour at RT, washed three times with PBS, and mounted in Slowfade mounting medium (Invitrogen) on slides for viewing.

For surface staining of MHCII, cells that were grown for 24 hours in the presence or absence of 100 μg/ml LPS were first washed three times with ice-cold PBS and were then incubated with primary antibody diluted in 1 × PBS/1 mg/ml BSA at 4° for two hours. Cells were then washed three times, five minutes each, with 1 × PBS/1 mg/ml BSA at 4° and were then incubated in secondary antibody diluted in 1 × PBS/1 mg/ml BSA at 4° for one hour. Cells were washed again and then fixed in 1% formaldehyde for 1 hour at 4° before washing with PBS and loading on slides. The confocal images of these cells were acquired using the same exposure and magnification. Quantitation of the intensity of surface MHCII labeling was done using Adobe Photoshop (Adobe Systems Incorporated, San Jose, CA).

Antibodies/dilutions used were Chicken anti-GFP/1:200 (Abcam, Cambridge, MA), Rat anti-Lamp1/1:1 (Developmental Studies Hybridoma Bank, Iowa City, IA), Mouse anti-lyso(bis)phosphatidic acid (LBPA)/1:1 [41], Rabbit anti-Mannose 6-Phosphate (CI-M6PR)/1:500, Rabbit anti-HRS/1:250 [42], and Rat anti-Mouse Major Histocompatibility Complex II (MHCII) – M5/114.15.2/1:5 (BD Biosciences, San Jose, CA) [56], or Mouse anti-Mouse Major Histocompatibility Complex II (MHCII) – 34-5-3/1:5 (BD Biosciences). For the 34-5-3 antibody, Fc Block was included with the primary antibody at a 1:50 dilution (BD Biosciences).

The percent co-localization is the number of GFP-ML1-stained structures that co-localized with markers for various compartments divided by the total number of GFP-ML1-stained structures in a section and multiplied by 100. The graphs show the average from sections of at least 20 different cells.

### Acridine Orange staining

RAW264.7, LS9, and LS10 cells were washed twice with PBS/0.5 mg/ml BSA and incubated in a 100 μm solution of Acridine Orange (AO, Sigma-Aldrich, St. Louis, MO) diluted in PBS/0.5 mg/ml BSA for 10 minutes at RT. Cells were then washed twice in PBS/0.5 mg/ml BSA and imaged immediately by confocal microscopy. All images were taken using the same exposure and magnification. Unstained cells were imaged as a control and did not show any background fluorescence under the conditions used to visualize the AO. We used ImageJ software to measure the mean intensity of staining of individual AO-stained compartments. At least 100 structures were measured for each strain to determine the means and standard deviations.

### Dextran-Oregon Green staining

RAW264.7, LS9, and LS10 cells were washed once with DMEM/F-12 medium and incubated in the same medium for 1 hour at 37°. Cells were then incubated in DMEM/F-12 medium containing 1 mg/ml dextran (MW-10,000)-Oregon Green 488 (Invitrogen) for 5 minutes at 37°. Cells were washed twice with DMEM/F-12 medium and chased for another hour at 37° before confocal microscopy. All images were taken using the same exposure and magnification. Unstained cells were imaged as a control and did not show any background fluorescence under the conditions used to visualize the dextran-Oregon Green 488. We used ImageJ software to measure the mean intensity of staining of individual dextran-Oregon Green 488-stained compartments. At least 60 structures were measured for each strain to determine the means and standard deviations.

### Bodipy-LacCer trafficking

Growing cells were washed twice with PBS and once with DMEM/F-12 medium. Cells were then incubated in DMEM/F-12 medium containing 10 mg/ml dextran (MW-10,000)-Cascade Blue (Invitrogen) for 4 hours at 37°. The staining solution was replaced with normal medium and the cells were left for 24 hours at 37°. Cells were washed twice with PBS and once with DMEM/F-12 medium and then left in DMEM/F-12 medium for 1 hour at 37°. Cells were then incubated in DMEM/F-12 medium containing 5 μM BODIPY-FL LacCer-BSA (Invitrogen) for 30 minutes at 37°. The staining solution was replaced with 2 ml of pre-heated DMEM/F-12 and the cells were left for 45 minutes or 90 minutes at 37°. To remove plasma membrane labeling after the chase, cells were back exchanged six times, 10 min each time, with 2 ml ice-cold DMEM/F-12/5% BSA and the plates were left on ice until all samples were ready for confocal microscopy. Unstained cells that were similarly treated, except for the addition of the dyes, were imaged as a control and did not show any background fluorescence under the conditions used to visualize the dextran-Cascade Blue or the BODIPY-FL LacCer-BSA.

### Hen Egg Lysozyme degradation assay

Growing cells were washed twice with PBS and once with DMEM/F-12 medium. Cells were then harvested in DMEM/F-12 medium and 10^7 ^cells were added to 100 mm plates. After 2 hours at 37°, cells were washed twice with 37°-pre-heated DMEM/F-12 medium. Cells were then incubated for 5 minutes in 37°-pre-heated Hen Egg Lysozyme (HEL) dissolved in DMEM/F-12 medium at 10 mg/ml. This solution was removed and the cells were washed three times with 37°-pre-heated PBS and 5 ml of 37°-pre-heated DMEM/F-12 was added to the cells. Cells were dislodged from the plates by scraping and clumps were broken up by pipetting. The 5 ml solution of cells was then added to 37°-pre-heated 15 ml tubes and incubated while mixing at 37°. This is time zero. At times 0 min, 30 min, 60 min, and 90 min, 1 ml of cells was removed and added to pre-chilled eppendorf tubes. Cells were spun down in the cold, washed once with ice-cold PBS, and resuspended in 150 μl of Western loading buffer (50 mM Tris pH 6.8, 10% glycerol, 4% SDS, 10 mM DTT, 0.01% Bromophenol Blue) preheated to 95°.

For Western analysis, 10 μl of each sample was used per lane. Each filter was cut horizontally such that the top half was probed using Rabbit anti-GAPDH (Cell Signaling Technology, Danvers, MA, 1:1000 dilution) and the bottom half was probed using Rabbit anti-HEL (Abcam, 1:5000 dilution). For antigen detection, we used Goat anti-Rabbit IgG secondary antibodies conjugated to HRP (Pierce, Rockford, IL, 1:50,000) and the Amersham ECL Advance Western Blotting Detection Kit (Amersham Biosciences, Pittsburgh, PA).

To quantitate the cellular levels of HEL over time, we used ImageJ to quantitate the intensities of the HEL and GAPDH bands on scanned images. For each lane, we divided the HEL intensity by the GAPDH intensity to normalize HEL levels relative to cellular protein. We then divided the normalized HEL number at the 30 min, 60 min, and 90 min time point by the number at the 0 min time point to determine the percent of cellular HEL relative to the 0 time point. We note that the reported differences between the cell lines may be more robust than shown here because of the non-linear nature of the HRP-based ECL detection assay.

The whole experiment was repeated twice to get averages and standard deviations.

### Microscopy

Confocal images were taken with a Nikon PCM 2000 using HeNe 543 excitation for the red dye and Argon 488 for the green dye or with a Zeiss-Meta 510 microscope.

## Abbreviations

Bovine Serum Albumin, BSA; Hen Egg Lysozyme, HEL; Lyso(bis)phosphatidic acid, LBPA; Major Histocompatibility Complex II, MHCII; Mannose 6-Phosphate Receptor, M6PR; Mucolipidosis Type IV, MLIV; Mucolipin-1, ML1; Room Temperature, RT. Wild type, WT.

## Authors' contributions

EGT and LS conceived and executed experiments in this study, and drafted and edited the manuscript. HD executed experiments in this study. HF supervised the study and edited the manuscript. All authors read and approved the final manuscript.
